# Packages of Care for Dementia in Low- and Middle-Income Countries

**DOI:** 10.1371/journal.pmed.1000176

**Published:** 2009-11-03

**Authors:** Martin J. Prince, Daisy Acosta, Erico Castro-Costa, Jim Jackson, K. S. Shaji

**Affiliations:** 1 Health Service and Population Research Department, Institute of Psychiatry, King's College London, London, United Kingdom; 2 Universidad Nacional Pedro Henriquez Ureña (UNPHU), Santo Domingo, Dominican Republic; 3 Centro de Pesquisa Rene Rachou/Fiocruz, Belo Horizonte, Minos Gerais, Brazil; 4 Alzheimer Scotland, Edinburgh, United Kingdom; 5 Medical College, Thrissur, India; London School of Hygiene and Tropical Medicine, United Kingdom

## Abstract

In the fifth in a series of six articles on packages of care for mental disorders in low- and middle-income countries, Martin Prince and colleagues discuss the treatment of dementia.

*This is the fifth in a series of articles highlighting the delivery of “packages of care” for mental health disorders in low- and middle-income countries. Packages of care are combinations of treatments aimed at improving the recognition and management of conditions to achieve optimal outcomes.*

**Summary Points**

- Two-thirds of people with dementia live in low- and middle-income countries (LMICs), where there are few services available and levels of awareness and help-seeking are low.
- After early diagnosis, the principal goals for management of dementia are optimising physical health, cognition, activity, and wellbeing; detecting and treating behavioural and psychological symptoms (BPSD); and providing information and long-term support to carers.
- Routine packages of continuing care should comprise diagnosis coupled with information, regular needs assessments, physical health checks, and carer support, and where necessary carer training, respite care, and assessment and treatment of BPSD.
- Care can be delivered by trained primary care teams working in a collaborative care framework. Continuing care with practice-based care coordination, and community outreach are essential components of this model.
- Efficient care delivery in LMICs involves integrating dementia care with that of other chronic diseases and community-support programs for the elderly and disabled.

## Introduction

Dementia is a chronic organic brain syndrome, characterised by progressive impairment of multiple cortical functions, including memory, learning, orientation, language, comprehension, and judgement. Diagnosis requires decline in cognitive function and independent living skills (Box 1) [1]. However, for carers and people with dementia, the behavioural and psychological symptoms of dementia (BPSD) affect most quality of life, are an important cause of carer strain [2], and a common reason for institutionalisation [3]. Alzheimer's disease, vascular dementia, dementia with Lewy bodies, and frontotemporal dementia are the most common dementia subtypes, but mixed pathologies may be the norm [4]. Some rare causes (subdural haematoma, normal pressure hydrocephalus, hypercalcaemia, and deficiencies of thyroid hormone, vitamin B12, and folic acid) can be treated. Otherwise, the progressive course of dementia cannot be altered, but symptomatic treatments and support can be helpful.

**Box 1. International Classification of Diseases 10 Criteria for Dementia and Commonly Occurring Features**

**Diagnostic criteria (ICD-10 Diagnostic Guidelines)**

“The primary requirement for diagnosis is evidence of a decline in both memory and thinking which is sufficient to impair personal activities of daily living. The impairment of memory typically affects the registration, storage, and retrieval of new information, but previously learned and familiar material may also be lost, particularly in the later stages. There is also impairment of thinking and of reasoning capacity, and a reduction in the flow of ideas. The processing of incoming information is impaired, in that the individual finds it increasingly difficult to attend to more than one stimulus at a time, such as taking part in a conversation with several persons, and to shift the focus of attention from one topic to another. If dementia is the sole diagnosis, evidence of clear consciousness is required. The above symptoms and impairments should have been evident for at least 6 months for a confident clinical diagnosis of dementia to be made” [1].

The diagnostic criteria do not convey a sense of the typical progression of the disorder:

**A person with mild dementia** has noticed deterioration in their memory for recent events. For example, they may forget that their daughter had visited the previous day. They also find it difficult to concentrate, think flexibly, plan, and take decisions. They are likely to feel bewildered, anxious and sad. They may become angry and defensive when others point out errors.

**A person with moderate dementia** has severe memory problems. Only early memories are retained. Recent events are not remembered, or rapidly forgotten. They may not know the day, date or time of day. They often do not know where they are. They cannot communicate clearly, having problems finding the right word and using the wrong words. They may hear voices or see things that are not there, and can develop false beliefs, for example that children are entering their house and stealing things. They are likely to be anxious, sad, bewildered, and can become agitated or aggressive.

**A person with severe dementia** has complete memory loss. They may no longer recognise their close family. They have severe speech difficulties or are unable to communicate. They may be apathetic and totally inactive, but at times can be agitated and verbally and physically aggressive. They cannot coordinate their physical movements; may have lost the ability to walk and feed themselves and have difficulty swallowing. They are likely to be incontinent.

Dementia mainly affects older people. Few cases start before the age of 65 y, after which prevalence doubles with every 5-y increase in age [5]. Globally, 24.3 million people are affected by dementia and 4.6 million new cases occur annually [6]. The prevalence of dementia is expected to double every 20 y, reaching 81.1 million by 2040, an increase of 100% in developed countries and of more than 300% in India, China, and their neighbours. Prevalence is lower in low- and middle-income countries (LMICs) than in high income countries (HICs) [6], perhaps because of underdetection of mild cases [7]. Nevertheless, most people with dementia live in LMICs—60% in 2001 rising to 71% by 2040 [6].

Dementia contributes 11.2% of years lived with disability among people aged 60 y and over, a higher proportion than stroke (9.5%), musculoskeletal disorders (8.9%), cardiovascular disease (5.0%), and cancer (2.4%) [8]. Its global cost is estimated to be US$317 billion, 77% of this total arising in HICs where formal sector care costs increase with disease progression, and institutionalization is the main cost driver [9]. Family care is more important in resource-poor countries, accounting for 56% of costs in low-income countries, 42% in middle-income countries, and 31% in HICs [9]. In a pilot study in 26 LMIC centers, carers were economically disadvantaged [10]. A fifth of carers had cut back on paid work, and paid carers were common, which added to the economic strain [10]. Compensatory benefits were practically nonexistent [10],[11].

In three qualitative studies in India, features of dementia were widely recognized and named [12]–[14]. However, dementia was perceived as normal ageing rather than as a medical condition. The consequences were limited help seeking [13] despite disability and carer strain [15], no structured training on the recognition and management of dementia, and no constituency to advocate for more responsive care services [14]. People with dementia were excluded from residential care [13]. Carers misinterpreted BPSD as deliberate misbehavior [14]. BPSD can also lead to stigma and blame attaching to the carers [2]. In India, likely causes of dementia were cited as “neglect by family members, abuse, tension and lack of love” [13].

In this article, we focus on the effective management of dementia in LMICs, reviewing the evidence on efficacy of interventions and their delivery derived from LMICs where possible. Given the paucity of relevant evidence from LMICs, we also cite systematic reviews and meta-analyses based on trials from HICs. On the basis of our review we propose a package of care—a combination of treatments aimed at improving the recognition and management of conditions to achieve optimal outcomes—for dementia.

## The Evidence on the Management of Dementia

The principal goals of management of dementia are: early diagnosis; optimization of physical health, cognition, activity, and wellbeing; detection and treatment of BPSD; and the provision of information and long-term support to carers. The evidence base for dementia care comes, overwhelmingly, from HICs (Table 1). All the studies discussed below refer to HICs, unless otherwise specified.

**Table 1:** The evidence in support of dementia management.

Outcome	Intervention	Evidence from HICs	Evidence from LMICs
**Identifying dementia**	Validity of screening measures	Systematic review of screening properties of brief cognitive test and informant screening assessments, validated in general practice or community settings [25]	Adapted versions of the Mini Mental State Examination in India, China, Brazil [20]–[22]; Community Screening Instrument for Dementia (CSID) [102],[103]
**Assessing needs**	Camberwell Assessment of Needs in the Elderly (CANE)	Development, reliability, and validity in UK, US, and Sweden [104]	Feasibility and reliability in Brazil, community study [94]
**Cognitive impairment**	ChEIs	Meta-analyses of RCTs of donepezil, rivastigmine, and galantamine in Alzheimer's disease [48]–[51]; Meta-analysis of RCTs of donepezil, rivastigmine, and galantamine for vascular cognitive impairment [57]	Small RCT of donepezil in Brazil [52]; Open label trials of galantamine in Brazil [53] and China [54]
	Memantine	Meta-analysis of RCTs of memantine [56]; Meta-analysis of RCTs of memantine for vascular cognitive impairment [57]	—
	Nonpharmacological interventions	Meta-analysis of RCTs of reminiscence therapy [47]; RCT of cognitive stimulation [44]; Meta-analyses of RCTs of cognitive training and rehabilitation [46] a	Small pilot trial of cognitive stimulation in Brazil [45]
**Behavioural symptoms**	Conventional antipsychotic drugs	Meta-analysis of RCTs of haloperidol [60]	—
	Atypical (second-generation) antipsychotic drugs	Meta-analyses of RCTs of risperidone, olanzapine, quetiapine, and aripiprazole for the treatment of BPSD, aggression, and agitation [61],[62]	—
	ChEIs	Meta-analysis of RCTs of ChEIs for the treatment of BPSD [51]	—
	Memantine	Meta-analyses of RCTs of memantine in severe dementia [56],[58]	—
	SSRI antidepressants	Small RCT of citalopram for the treatment of agitation [73]; Meta-analysis of RCTs of trazodone [72]a; RCT of sertraline [74]a	—
	Anticonvulsants	Two small RCTs of carbamezepine [76],[77]	—
	Environmental/sensory interventions	Meta-analysis of RCTs of massage and light touch [81]; Meta-analysis of the efficacy of aroma therapy [105]; Meta-analysis of RCTs of Snoezelen (multisensory stimulation) [79] a; Meta-analysis of RCTs of light therapy [78] a	—
**Depression**	Antidepressant pharmacotherapy	Meta-analysis of RCTs of antidepressants to treat depression in dementia [70] a	—
	Carer interventions	Meta-analyses of RCTs and nonrandomised trials of carer interventions for improving mood in the care recipient [84],[86]	—
**Psychosis**	Atypical antipsychotic drugs	Meta-analyses of RCTs of atypical antipsychotic drugs for the treatment of psychosis in Alzheimer's disease [61],[62]	—
**Carer strain, subjective wellbeing, and psychological morbidity**	Carer interventions	Meta-analyses of controlled studies (including nonrandomised trials) of carer interventions in general [86] and the specific effects of psychoeducational, interventions, CBT, counselling, and respite care [84]; Meta-analysis of RCTs of respite care [87] a	Two small RCTs of a carer education and training intervention in Goa, India [89] and in Moscow, Russia [90]
**Institutionalisation**	Carer intervention	Meta-analysis of controlled studies (including nonrandomised trials) of carer interventions in preventing or delaying institutionalisation [84],[88]	—

a The evidence from these trials and meta-analyses is inconclusive, usually because of a combination of the following factors: small trials; small number of trials; flawed methodology (see text for details).

SSRI, selective serotonin reuptake inhibitor.

### Detection and Diagnosis of Dementia

Many cases of dementia, particularly in LMICs, go undetected,in part because of lack of awareness. Awareness of this disorder can be boosted by dissemination of information from governments, health care providers, and media. Help-seeking can be encouraged by improved case-finding. In India and Brazil, for example, community health care workers could, with a few hours training, identify dementia in the community with a positive predictive value of 66% [16],[17]. Population screening for dementia is not considered cost-effective even in HICs [18]. Selective screening can be accomplished by cognitive testing or by informant report of cognitive and functional decline. The Mini-Mental State Examination [19] is widely used in HICs, and adapted versions have been developed for use in many LMICs [20]–[22]. However, this assessment takes 10 min to administer and is prone to educational and cultural bias [23],[24]. A systematic review of brief screening assessments identified three tests (the General Practitioner Assessment of Cognition, Mini-Cog, and Memory Impairment Screen) that took less than 5 min to administer and were considered suitable and valid for routine use in general practice [25]; none of these tests has been validated in LMICs.

Dementia diagnosis requires cognitive testing, clinical interview, informant interview, and physical examination. The 10/66 Dementia Research Group's culture- and education-fair diagnostic protocol is validated in many LMICs [26],[27], but requires adaptation for clinical use. Practice guidelines in HICs [18] advocate a routine “dementia screen” to exclude treatable causes, which includes haematology, biochemistry, thyroid function tests, vitamin B12 and folate levels. The feasibility and cost-effectiveness of this approach needs to be tested in LMICs.

Finally, a recent systematic review based on evidence collected in HICs identified good practice for disclosing dementia diagnosis. This practice should include: preparation; integrating family members; exploring the patient's perspective; disclosing the diagnosis; responding to patient reactions; focusing on quality of life and wellbeing; planning for the future; and communicating effectively [28]. The person assessed should be asked if they, and/or others wish to be told the diagnosis. If so, they should be given information about the signs, symptoms, and course of dementia, available treatments, care and support services.

### Physical Assessment

To avoid missing underlying conditions, a physical assessment is recommended before specific treatments for BPSD are initiated [29], and should be a regular part of care for all patients. BPSD may sometimes be caused by pain, constipation, and urinary tract infection, although such associations are not always observed [30]. Pain is common and poorly controlled in severe dementia [31]. Hearing and visual impairment impede communication and exacerbate disorientation, and deafness predicts rapid cognitive decline [32],[33]. Visual and auditory impairment can be associated with hallucinations and delusions [34],[35]. Studies indicate that all these impairments are overrepresented in people with cognitive impairment [36]–[38].

There have been very few trials of the effects of physical assessments and interventions on the course of dementia. In a randomised controlled trial (RCT), pain assessment among nursing home residents with dementia was associated with increased analgesic use, reduced pain, and improvements in staff morale [39]. Uncontrolled studies show that audiological assessment is feasible, that hearing aids can be beneficial [40], and that referral to an optician to improve visual acuity may reduce visual hallucinosis [34]. Nutrition is often impaired because of apathy, aversive feeding behaviours, poor dental health, and dysphagia. Although difficult to sustain, nutritional supplementation improved nutrition among nursing home residents [41]; nutritional education for carers had the same effect in the community [42]. A “vascular care” secondary preventive intervention for people with dementia and cerebrovascular disease (part of current good practice guidelines [18]) that addressed hypercholesterolemia, hypertension, smoking, obesity, exercise, and micronutrient deficiency had no impact on subsequent cognition, disability, or institutionalisation [43].

### Psychological Treatments

A well-conducted RCT of cognitive stimulation (reality orientation, games, discussions based on information processing rather than knowledge) conducted in the United Kingdom as a group intervention [44], and a small pilot trial from Brazil [45], suggest that cognitive benefits from this intervention are similar to those for cholinesterase inhibitors (ChEIs). More specific cognitive training produced no benefits [46]. Cognitive rehabilitation, an individualised therapy designed to enhance residual cognitive skills and cope with deficits, showed promise in uncontrolled case series undertaken in HICs [46]. A meta-analysis of four trials of reminiscence therapy (the discussion of past activities, events, and experiences) [47] provides evidence for short-term improvement in cognition, mood, and carer strain, but the quality of these trials was poor.

### Pharmacological Treatments

Targets for pharmacological treatment include cognitive impairment, behavioural symptoms (agitation and aggression), and psychological symptoms (depression, anxiety, and psychosis).

There is a strong evidence base for the efficacy of ChEIs (donepezil [48], rivastigmine [49], and galantamine [50]). The use of each of these drugs is associated with modest and comparable improvements in cognitive function, global clinical state, and activities of daily living [51]. The evidence base for ChEIs from LMICs is limited to one small RCT of donepezil in Brazil [52] and open-label trials of galantamine in Brazil [53] and China [54]. The efficacy of this class of drugs in severe dementia is unclear, although useful cognitive benefits were identified for galantamine [55]. A fourth drug for the treatment of cognitive impairment, memantine, has a different mode of action, and is well tolerated, but evidence for its efficacy is limited to people with moderate to severe dementia [56]. ChEIs and memantine are less efficacious in vascular dementia than in other forms of dementia [57]. Their efficacy for the treatment of disturbed behaviour is not established; manufacturer-sponsored licensing trials [51] and post hoc analyses [58] indicate small improvements that have not been confirmed in independent trials [59] and meta-analyses [56].

Meta-analyses of RCTs of haloperidol [60] and atypical antipsychotic drugs for the treatment of agitation [61] and BPSD [62] indicate small treatment effects, most evident for aggression [62],[63]. Atypical antipsychotic drugs have also been widely prescribed for psychosis in dementia, but a meta-analysis of their efficacy indicated that only aripiprazole had a statistically and clinically significant effect [62]. Use of these drugs in dementia is associated with an increased risk of death and cerebrovascular adverse events [62],[64]–[68].

The benefits of antidepressant treatment in older people are clear [69], but a meta-analysis of their efficacy in people with dementia was inconclusive [70]. Only two small trials were included in this meta-analysis, one of which suggested a benefit of sertraline for some depression outcomes [71]. Antidepressants have also been proposed for the treatment of BPSD. A meta-analysis of two small RCTs of trazodone was inconclusive [72]. Citalopram showed efficacy over placebo for the treatment of agitation in one small RCT [73], while sertraline showed no benefit on any primary behavioural endpoint [74].

A systematic review of trials of anticonvulsants to treat BPSD found sodium valproate to be ineffective [75]. Carbamezepine may be more promising with large benefits noted for global clinical outcomes and agitation in one small parallel group trial [76] and more marginal effects in a small pilot trial [77].

### Sensory Therapy

Various sensory therapies have been proposed as treatments for BPSD but the evidence base for this approach is small and limited by the poor quality of the trials. Current evidence does not support the use of bright light therapy [78] or multisensory stimulation [79]. One small RCT of aromatherapy suggested considerable benefit across a range of behavioural outcomes [80]. Another small but well-conducted trial suggested that hand massage may be effective in reducing agitation [81],[82]. More evidence is required to confirm efficacy, exclude the possibility of harm, and define the optimal content and mode of administration of sensory therapies in both HICs and LMICs, where the approach is untested.

### Carer-Focused Interventions

A large literature attests to the benefits of carer interventions in dementia [83]. These include: psychoeducational interventions, often including carer training; psychological therapies such as cognitive behavioural therapy (CBT) and counselling; carer support; and respite care. Many interventions combine several of these elements. There are several systematic reviews and meta-analyses of these interventions [84]–[88]; all of the constituent trials in these studies were conducted in HICs, and many were nonrandomised [84]. Outcomes studied include carer strain, depression, and subjective wellbeing; behaviour disturbance and mood in the care recipient; and institutionalisation. Most carer-focused interventions seemed to reduce carer strain and depression, CBT having the largest impact on depression [84]. Psychoeducational interventions required the active participation of the carer (for example, in role-playing activities) to be effective [84]. Carer support increased carer wellbeing but no other outcomes [84]. For respite care, three methodologically flawed RCTs showed no benefit on any outcome [87]. However, nonrandomised studies suggest that respite care significantly reduces carer strain and psychological morbidity [84]. Interventions targeting the carer may also have small but significant beneficial effects upon the behaviour of the person with dementia [84]. A systematic review of ten RCTs indicated a 40% reduction in the pooled odds of institutionalisation [88]; the effective interventions were structured, intensive, and multicomponent, offering a choice of services and supports [84],[88]. Two small trials in LMICs of a brief carer education and training intervention, one from India [89] and one from Russia [90] indicated much larger treatment effects on carer psychological morbidity [89] and strain [90] than typically seen for such interventions in HICs.

## Delivery of Effective Interventions

The mechanisms by which effective dementia care treatments may be delivered in LMIC settings are summarized in Table 2.

**Table 2:** Delivering dementia care treatments.

Step	How	By Whom	In What Settings
**Increasing demand**	Improve awareness among general population, carers, health professionals	Alzheimer's associations, other chronic disease NGOs, health professionals, media, government	Schools
	Combat stigma	—	Public arena
	Provide age-appropriate, accessible services	—	General practice, primary care
	Provide disability pensions for people with dementia, and carer benefits	—	Professional education institutions
**Increasing capacity of health care teams**	Training of community, primary, and secondary general health care providers (diagnosis, needs assessment, health checks, care package)	Medical, nursing, and rural health schools	Primary care
	Paradigm shift to chronic, continuing care	Dementia specialist clinicians	Secondary general health care
	Support and supervision from specialists	—	—
**Increasing recognition**	Community-based case finding [16],[17]	Community health care workers	Community
	Selective screening in primary and secondary care [25]	Nonspecialist clinicians	Primary care
	Specialist centers	Dementia specialist clinicians	Secondary general health care
	—	—	Memory clinics
**Adapting treatments to increase acceptability or reduce cost**	Integrate dementia prevention and care into new chronic disease programs [93]	Community health care workers to deliver carer interventions and act as care coordinators [100]	Community
	Provide outreach (home-based assessment and care)	Nonspecialist clinicians to diagnose dementia, optimize physical health, assess needs, and plan community support	Primary care
	Integrate long-term care and support interventions into programs for all dependent elderly	Carers and other volunteers	Secondary general health care
	Ensure carer interventions are culturally appropriate [89]	—	—
	Provide group interventions for carers [83]	—	—
	Develop and use cheaper generic versions of ChEIs and SSRI antidepressants, where available and indicated	—	—
**Practice-based programs to deliver effective treatments**	Train primary care staff as dementia and elder care case managers [100]	Primary care case managers	Primary care
	Establish collaborative care [100] with involvement of specialist teams	Primary care clinicians	—
	—	Primary care health care workers	—
	—	Dementia care specialist	—
**Community-based programs to deliver effective treatments**	Train carers and establish and support groups	Community health care workers	Community
	Home outreach and individualized intervention	Peer (carer-to-carer) support and training	—
	Respite at home and homecare	Community volunteers	—
**Addressing impact of the disorder on other health/social outcomes**	Carer interventions	Government social welfare agencies	Community
	Carer benefits	Community health care workers	Primary care
	Respite care and homecare programs	Case managers	—
	Disability benefits and social pensions for older people [106]	—	—
	Regular physical health checks	—	—
	Nutritional interventions	—	—

SSRI, selective serotonin reuptake inhibitor.

### Interventions to Increase Demand for Services

Alzheimer's Disease International has identified raising awareness of dementia among the public, carers, and health workers as a global priority, with an increase in demands for services as one of the intended benefits [91]. Awareness can be raised in several ways. The establishment of a critical mass of informed carers can assist awareness-raising, provide advice and support to families, and work with Alzheimer's disease associations to lobby for more services that better meet the needs of carers. Community solidarity can also effect change through support for health and social welfare policies based on equity and justice. Aware communities can provide support and reduce stigma and exclusion. Policymakers can be held to account by media campaigns and advocacy from committed NGOs. In HICs, awareness is growing rapidly, with the media playing an important role [92]. Media in LMICs can be receptive to these stories, but efforts are required to alert them to the importance of ageing and dementia, and to build their capacity to report the problem [92].

Intergenerational solidarity can be promoted through awareness-raising among children and young adults. In many LMICs, many people with dementia live in multigenerational households with young children, and children or children-in-law are the most frequent carers for people with dementia [10], and the most likely to initiate help-seeking. Finally, in LMICs the provision of disability pensions and carer benefits will inevitably increase requests for diagnostic assessment. Importantly, however, efforts to increase awareness must be accompanied by health system and service reform, so that help-seeking is met with a supply of better-prepared, more responsive services.

### Interventions to Improve the Capacity of Health Care Teams

Primary health care services in LMICs often fail older people because they are clinic-based and preoccupied with simple curative interventions [13]–[15]. A paradigm shift is needed to encompass continuing care and support as part of a wider chronic disease strategy. Given the frailty of many older people with dementia, there is also a need for outreach to assess and manage patients in their own homes. The World Health Organization (WHO) Innovative Care for Chronic Conditions framework [93] proposes that the delivery of care for chronic conditions can be improved through a dialogue to build commitment for change, extended and regular health care contact, a multisectoral approach, care centered on patients and families, support for patients in the community, and an emphasis on prevention. Dementia care should be an essential component of such chronic disease care strategies. Training of nonspecialist health professionals should focus on case-finding and on conveying the diagnosis to patients and carers together with information and support, needs assessment, and carer training and support. Training can be service-based as well as through changes to the medical and nursing school, public health, and rural health curricula. Medical and community care services should be planned and coordinated to respond to the increasing need for support as the disease progresses.

### Interventions to Improve Recognition

The focus here should be first upon case-finding by community health workers [16],[17], and then on selective screening coupled with simplified “quasi-diagnostic” algorithms and needs assessments [94] by nonspecialist clinicians. More research is needed to identify the most feasible and valid methods.

### Interventions to Increase the Acceptability and Reduce the Costs of Treatments

At present, the costs of ChEIs are reimbursed in some Latin American countries and Chinese provinces, generic ChEIs are available in India, and Huperzine A, a cheap plant extract with similar properties to ChEIs is used in China [95]. In what is a very active and promising field for drug development [96], any new agent with radical disease-modifying properties will be very expensive, which raises important ethical and practical challenges to securing equitable access. An international effort leveraged by grass roots advocacy (similar to that mounted for antiretroviral treatment for HIV) might be required to secure affordable supplies for people with dementia living in LMICs. More positively, costs of primary care and community interventions can be minimised through task-shifting to nonspecialist and paraprofessional staff and integration with more broadly based chronic disease [93], disability, and elder care programmes [97],[98]. In India a community-based palliative care program has been successfully extended to include dementia care [99].

### Practice-Based Programs to Deliver Effective Treatment

Most LMICs have insufficient specialists to provide national frontline dementia services. Diagnosis and needs assessment can be conducted in primary care, although increased specialist input to help with advice, inpatient or outpatient review of refractory BPSD, and respite care would be desirable. Routine physical reviews within practice-based programs are important but research is needed into the cost-effectiveness of haematological and biochemical screening, and the feasibility and effectiveness of interventions to improve hearing, vision, nutrition, and continence. A commitment to continuing care is essential, with regular review and physical health and needs assessments. Practice-based case managers can coordinate this process; this collaborative care model reduced BPSD and carer strain in an RCT in the US [100].

### Community-Based Programs to Deliver Effective Treatments

Carer-support programs can be delivered individually or in groups by community health workers or by experienced carers. Carer strain, whether or not associated with BPSD, should trigger more intensive intervention that includes psychological assessment and depression treatment for the carer [101], respite, and carer education and training. Such interventions could be incorporated into horizontally constructed community-based programs that address the generic needs of frail, dependent older people and their carers, whether these needs arise from cognitive, mental, or physical disorders.

## Packages of Care for Dementia in LMICs

On the basis of our review, we have proposed a package of care for LMICs that considers the availability of resources for the detection, diagnosis, and treatment of dementia in this setting. In Table 3, the main features of this package are compared with typical recommendations for well-resourced HIC settings. To improve care for dementia in LMICs, we propose that the basic package of care should focus upon diagnosis coupled with information, regular needs assessments, physical health checks, and carer support. This package should be extended to include carer training, respite care, and assessment and treatment of BPSD where possible. We suggest that that care can best be delivered by trained primary care teams, with a paradigm shift towards chronic continuing care and community outreach. Practice-based care coordinators are an essential component of this package, ideally working within a collaborative-care framework. Finally, we note that care delivery will be more efficient when integrated with that of other chronic diseases, and more broadly based community-support programs for the elderly and disabled.

**Table 3:** Packages of care for dementia.

Low Resourced Settings	High Resourced Settings [107]
Public information campaigns	Public information campaigns
Community case-finding; Primary care diagnosis and needs assessment	National network of specialist memory services, for diagnosis and as entry point into care pathway
Provision of information for people with dementia and carers	Provision of information for people with dementia and carers
Continuity of support, coordinated by primary care case manager	Continuity of support, coordinated by specialist case manager
Regular case review with reassessment of needs; Specialist consultation and referral where available	Integrated and continuing health and social care by multidisciplinary specialist care team
Cautious use of antipsychotic drugs and other experimental treatments for BPSD, not as first line treatment, and preferably initiated and reviewed by specialists	Cautious use of antipsychotic drugs and other experimental treatments for BPSD, not as first line treatment, and initiated and reviewed by specialists
Anticholinesterase drug treatment for cognitive impairment is unlikely to be seen as a cost-effective priority within most resource-poor systems	Anticholinesterase drugs for memory impairment, when considered cost-effective, with specialist initiation and review
Carer-support groups; Individualised community-based carer interventions and support	Comprehensive carer's strategy for people with dementia; Community personal support
Promote access to general health care	Effective care in general hospitals, intermediate care and care homes. Effective end of life care

## Abbreviations

BPSD: behavioural and psychological symptoms
ChEI: cholinesterase inhibitor
HIC: high income country
LMIC: low- and middle-income country
RCT: randomised controlled trial
